# Improved glycaemic control in patients with type 2 diabetes has a beneficial impact on NAFLD, independent of change in BMI or glucose lowering agent

**DOI:** 10.1016/j.numecd.2022.12.010

**Published:** 2023-03

**Authors:** Santo Colosimo, Garry D. Tan, Maria Letizia Petroni, Giulio Marchesini, Jeremy W. Tomlinson

**Affiliations:** a School of Nutrition Science, University of Milan, Milan, Italy; b Oxford Centre for Diabetes, Endocrinology and Metabolism, NIHR Oxford Biomedical Research Centre, University of Oxford, Churchill Hospital, Oxford, OX3 7LE, UK; c Department of Medical and Surgical Sciences, University of Bologna, Bologna, Italy

**Keywords:** NAFLD, T2DM, Fatty liver Index, Fib-4, Glucose control, GLP-1RA, SGLT2i, DPP-IVi

## Abstract

**Background and aim:**

The current focus of the treatment of Non-Alcoholic Fatty Liver Disease (NAFLD) is lifestyle intervention with the aim of significant weight loss alongside aggressive cardiovascular risk reduction. NAFLD is tightly associated with type 2 diabetes (T2D) and obesity. In patients with T2D, glucose lowering agents that promote weight loss have shown a beneficial impact on NAFLD. However, it remains unclear as to whether glucose lowering can improve NALFD in patients with T2D, independent of weight loss.

**Methods and results:**

In a retrospective analysis of data from 637 people with T2D, we examined the longitudinal impact of optimizing glycaemic control with DPP-IV inhibitors, GLP-1RAs and SGLT2 inhibitors on Fatty liver index (FLI) and Fibrosis score 4 (Fib-4) adjusting for changes in BMI and choice of glucose lowering regimen over a 12-month period.

Multiple linear regression analysis demonstrated a significant correlation between the change in glycated haemoglobin and change in FLI after adjustment for change in BMI, age, sex, and drug class (R = 0.467, p = 0.031). The greatest reduction in FLI was observed in patients with the largest reduction in glycated haemoglobin (p < 0.0001). The probability of improvements in FLI with optimization of glycaemic control was similar with all 3 glucose lowering agents, despite differences in weight reduction. Similar relationships were observed examining the changes in glycaemic control and Fib-4.

**Conclusions:**

Improvements in glucose control that are independent of weight loss are associated with improvement in NAFLD and should form an integral part of the management patients with co-existent NAFLD and T2D.

## Highlights

• Lifestyle changes with weight loss improve NAFLD.
• There is a lack of data demonstrating the longitudinal impact of improved glucose control on NAFLD in patients with T2D.
• This study shows that a reduction in HbA1c is associated with a decrease in liver fat measured using surrogate markers.
• Liver fat improvement is independent of weight loss or specific glucose lowering agent.
• Out-patient clinic management of NAFLD should include optimization of glucose control in patients with coexistent T2D.

## 1 Introduction

### 1.1 Relevance

Non-alcoholic fatty liver disease (NAFLD) is a common disorder affecting 25% of the global population. It is often regarded as the hepatic manifestation of the metabolic syndrome and is tightly associated with obesity and type 2 diabetes (T2D) [1]. It has an associated morbidity and mortality through liver-specific causes, but more importantly through adverse cardiovascular outcomes. There are no approved pharmacological therapies, and the current mainstay of treatment relies upon significant weight loss and aggressive cardiovascular risk reduction.

### 1.2 Review of the literature

A metanalysis including data from 8 studies in 373 patients, of which 39% of patients had T2D, has shown that a 7–10% reduction in body weight is associated with histological improvement in NAFLD as assessed by the NAFLD activity score [2]. In addition, there is a rapidly developing body of evidence demonstrating the beneficial impact of glucose lowering agents in the management of patients with NAFLD [[3], [4], [5]] that have been trialed in patients with and without T2D. Almost all of these agents improve glycaemic control alongside inducing weight loss. Insulin sensitizing drugs including metformin and pioglitazione have been investigated as possible treatments for NAFLD [2]. Whilst metformin did not provide any histological benefit, pioglitazone treatment decreased hepatic steatosis and inflammation. However, its use in the clinical practice is limited due to concerns over adverse effects such as weight gain, bone fracture risk and fluid retention. Glucagon-like peptide 1 receptor agonists (GLP-1RA) have been shown to reduce liver fat content and weight in patients with and without T2D [4,6]. GLP-1RAs restore euglycaemia through various mechanisms including enhancing glucose-stimulated insulin secretion, inhibition of glucagon secretion, increased energy disposal, delay in gastric emptying and enhancement of satiety [7]. The LEAN study used Liraglutide and was the first of a series of trials testing the effectiveness of GLP-1RAs in the management of NAFLD demonstrating histological improvement [6]. Semaglutide, a long-acting GLP1-RA analog, provided similar benefits in terms of NASH resolution, but failed to meet the secondary endpoint of fibrosis reduction [4]. The sodium-glucose co-transporter-2 inhibitors (SGLT2i) are widely used to treat T2D with evidence of weight loss and cardiovascular risk reduction in addition to glucose lowering. Liver biopsy studies to investigate their role as a potential treatment for NAFLD have not been reported, but consistent improvements in liver chemistry have been observed [[8], [9], [10], [11]]. More recently there has been much interest in the hepatic effects of Tirzepatide, a GLP-1/GIP dual-agonist approved in USA for T2D treatment, that promotes reduction of liver fat content alongside significant weight loss in subjects with T2D [12]. This latter study is one of the few to date that have looked at longitudinal changes in glycated haemoglobin and determined its impact on hepatic steatosis [[12], [13], [14]]. In these studies, insulin was used as standard care treatment for the control group and hepatic steatosis improved as did glucose control, but surprisingly, there was no reported change in BMI.

Retrospective, cross-sectional data has suggested a relationship between worsening NAFLD severity (assessed with biomarkers, imaging or histology) and less tight glycaemic control [[15], [16], [17]]. Therefore, a critically important and unanswered question remains whether optimization of glycaemic control can improve NAFLD, independent of changes in weight in patients with type 2 diabetes.

### 1.3 Aims

Using the data from an observational study [18], we have undertaken a post-hoc analysis to determine whether changes in hepatic steatosis (fatty liver index) and risk of advanced fibrosis (Fib-4) are related to changes in glycaemic control, and if they occur independently of alterations in BMI and type of glucose-lowering agent.

## 2 Method

### 2.1 Population

We undertook a retrospective analysis of the clinical records of all patients with T2D attending our metabolic Unit who were consecutively initiated on therapy with DPP-IVi, GLP-1RA, SGLT2i glucose lowering agents in the period between 2014 and 2017. Data record and collection was facilitated by the implementation of a new digital registration system. A rigorous monitoring of these patients with enhanced consultations frequency and thorough bloods and urine testing was recommended by the Italian Medicines Agency (AIFA) as one of the criteria to be fulfilled in order to grant refunded drug prescription by the national healthcare service. This facilitated enhanced clinical data collection that was consistent across the entire study population.

The indication to initiate novel antidiabetic drug treatment was prompted by poor glycaemic control and the decision to allocate specific therapy was determined by patients’ preferences and adherence, comorbidities, hypoglycaemia risk and cardiovascular risk.

All patients who were on metformin continued to take it without dose alteration. Patients who had discontinued treatment for adverse events or failed to attend the clinic at follow-up appointment were excluded from the analysis. As part of the standard care that was provided, all patients received education for lifestyle change aiming at improved glucose control and weight reduction. Follow up evaluation was performed for all patients at 12 months from treatment initiation.

As internal audit, and due to the retrospective nature of the study, no informed consent was required according to the Italian law. Ethical approval was granted by the hospital trust ethical committee.

### 2.2 Measurements

As part of a local program of standardization of biochemical assays, from 2008 onwards, the metropolitan area of Bologna activated a centralized clinical laboratory hub to serve all the local hospital trusts. Liver ultrasound was periodically performed as a routine screening for patients with T2D. In addition, fatty liver index (FLI) and Fibrosis score 4 (Fib-4) were routinely calculated as surrogate marker of fatty liver and fibrosis respectively.

The fatty liver index [19] and Fib-4 [20] were calculated using the equations below:

$$FLI=\left(e^{0.953\times loge\left(triglycerides\right)+0.139\times BMI+0.718\times loge\left(GGT\right)+0.053\times waistcircumference-15.745}\right)/\left(1+e^{0.953\times loge\left(triglycerides\right)+0.139\times BMI+0.718\times loge\left(GGT\right)+0.053\times waistcircumference-15.745}\right)\times 100$$

$$\left[\mathrm{FIb}-4\right]=Age\times AST/\left[\mathrm{Platelet}\times {\mathrm{ALT}}^{1/2}\right]$$

$$\left[Platelet]=\left(10^{9}/L\right),[AST,ALT,GGT]=\left(U/L\right),[Triglyceride]=(mg/dL),[Waist\;circumference]=cm,[BMI]=(kg/m^{2}\right)$$

These scores were preferred over other biomarkers as they do not consider glucose control indicators and are independent of diabetes.

For the FLI, values under 30 are considered to be at low risk of steatosis, between 30 and 59 indeterminate and over 60 to be at high risk [19]. Cut-offs for Fib-4 were used according to the most recent literature guidance and are reported as follows: <1.30 low risk, between 1.30 and 2.67 indeterminate, and >2.67 high risk [21].

### 2.3 Data management, statistical approach and analysis

Anonymized data were extracted from hospital records. Patients were stratified according to their glycated haemoglobin response to new therapy initiation (DPP-IV inhibitors (DPP-IVi), GLP-1 receptor agonists (GLP-1RA) or SGLT2 inhibitors (SGLT2i)). Those patients with a glycated haemoglobin reduction of at least 11 mmol/mol (1%) over the 12-month duration of the study were defined as ‘*good glycaemic responders’*. Those with a reduction in glycated haemoglobin of between 1 and 10 mmol/mol as ‘*moderate glycaemic responders’* and those with no change or an increase in glycated haemoglobin as ‘*glycaemic non-responders’.*

A descriptive analysis was initially performed, followed by ANOVA for baseline data and paired t-test for time-course comparison of each variable. Bonferroni correction was applied to multiple comparisons.

Association between baseline variables were analyzed by multiple linear regression. The effects of glycaemic control on FLI and Fib-4 were measured by means of logistic regression analyses adjusted for confounders, namely age, sex, drug classes and change in BMI.

Statistical analysis was performed using Jamovi (version 1.6, The jamovi project, Sydney, Australia) and replicated with Prism GraphPad (version 8.0.0 for Mac, GraphPad Software, San Diego, California USA).

## 3 Results

Baseline characteristics of the population had been previously reported [18]. Briefly, 637 consecutive patients were recruited and included in the analyses. 385 were male (60%) and mean (± standard deviation) age was 61.6 ± 10.2 years; 282 (44%) fell into the *good glycaemic responders* group, 232 (36%) into the *moderate glycaemic responders*, and 123 (20%) into the *glycaemic non-responders*. The *good glycaemic responders* cohort had elevated HbA1c, ALT and FLI compared to the other groups. The baseline characteristics of each group are presented in Table 1.

**Table 1:** Baseline characteristics of the study population (data are mean ± standard deviation). Multiple comparisons with ANOVA, p-value corrected with Bonferroni.

	Total (n = 637)	Good glycaemic responders (n = 282)	Moderate glycaemic responders (n = 232)	Glycaemic non-responders (n = 123)
Sex – M (n (%))	385 (60)	160 (57)	143 (62)	82 (67)
Age (years)	61.6 ± 10.2	60.3 ± 10.6	63.0 ± 10.0∗	62.0 ± 9.3
HbA1c (mmol/mol)	67.0 ± 13	73.8 ± 13	62.7 ± 10∗	59.9 ± 10 ^#^∗
HbA1c (%)	8.28 ± 1.21	8.9 ± 1.2	7.89 ± 0.93∗	7.59 ± 0.99∗^#^
BMI (kg/m^2^)	35.3 ± 7.4	36.1 ± 7.6	34.6 ± 7.0∗	34.8 ± 7.6
Obesity (%)	77	80	73	75
Waist Circumference (cm)	110 ± 14	112 ± 15	109 ± 14	109 ± 15
Fasting Blood Glucose (mg/dL)	168 ± 49	179 ± 56	161 ± 39∗	157 ± 42∗
Fasting Blood Glucose (mmol/L)	9.3 ± 2.7	9.9 ± 3.1	8.9 ± 2.2	8.7 ± 2.3
ALT (μ/L)	34.1 ± 21.7	36.9 ± 24.5	32.1 ± 20.0∗	31.4 ± 16.7∗
GGT (μ/L)	41.8 ± 18.7	43.8 ± 23.3	40.5 ± 15.8	39.9 ± 9.2
Platelets (× 10^9^/L)	213 ± 43	212 ± 44	212 ± 42	212 ± 41
Fib-4	1.38 ± 1.27	1.37 ± 0.72	1.44 ± 0.69	1.30 ± 0.47
FLI	84.3 ± 18.2	87.4 ± 15.6	82.3 ± 19.2∗	81.0 ± 20.8∗
SBP (mmHg)	133 ± 16	133 ± 16	134 ± 17	133 ± 16
DBP (mmHg)	82 ± 13	83 ± 14	83 ± 12	82 ± 10
Creatinine (μmol/L)	76.9 ± 23.9	75.1 ± 28.3	77.8 ± 20.3	80.4 ± 20.3
Total Cholesterol (mmol/L)	4.65 ± 1.19	4.78 ± 1.29	4.58 ± 1.09	4.5 ± 1.14
Ultrasound evidence of steatosis (n (%))	423 (81)	206 (87)	130 (75)	78 (78)
Use of Statin and/or Ezetimibe (%)	56	50	60	60
DPP-IVi/GLP-1RA/SGLT2i (n)	104/338/195	38/152/92	48/114/70	18/72/33

∗p < 0.05 vs. good glycaemic responders.

^#^p < 0.05 vs. moderate glycaemic responders.

*Abbreviations*: HbA1c, glycated haemoglobin, BMI, body mass index, ALT, alanine-transaminase, GGT, gamma-glutamyl transferase, FLI, fatty live index, Fib-4, Fibrosis-4 score, SBP, systolic blood pressure, DBP, diastolic blood pressure, U.S., ultrasound, DPP-IVi, dipeptidyl-preptidase 4 inhibitors, GLP-1RA, glucagon-like peptide 1 receptor agonists, SGLT2i, sodium-glucose cotransporters 2 inhibitors.

**Figure 1:**
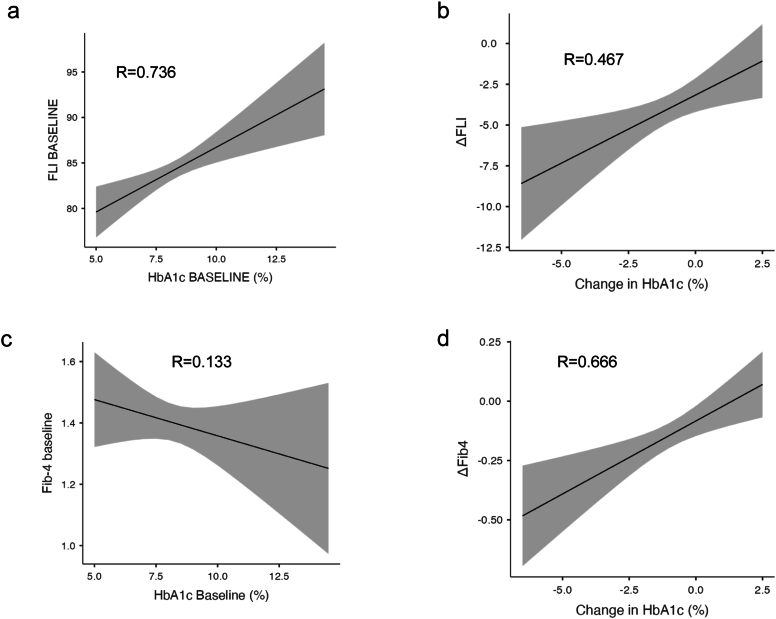
(a) Positive correlation between fatty liver index (FLI) and glycated haemoglobin (corrected for body mass index, BMI) at baseline. The change in both FLI (b) and FIB-4 (d) are associated with the change in glycated haemoglobin after correcting for change in BMI. Correlation between Fi-4 and HbA1c at baseline (c). Grey shaded area is 95% C.I.

At baseline, prior to new drug initiation, FLI correlated with glycated haemoglobin, even after adjustment for BMI (R = 0.736, overall model test p = 0.001, ANOVA for HbAc1 at baseline p = 0.001) (Fig. 1a).

After 1-year treatment with SGLT2i, GLP-1RA or DPP-IVi, *good glycaemic responders* observed a reduction in HbA1c from 8.9 to 6.86% (74–51 mmol/mol), *moderate glycaemic responders* from 7.89 to 7.39% (63–57 mmol/mol) and *glycaemic non-responders* had an increase from 7.59 to 8.23% (59–66 mmol/mol) (Table 2). BMI significantly decreased in all groups compared to baseline, with the greatest reduction observed in the *good glycaemic responders* group. Waist circumference was significantly reduced at different extents in all groups. Total cholesterol was significantly decreased in *good glycaemic responders* and *moderate glycaemic responders* and ALT only in *good glycaemic responders*. Across all groups, a small but significant reduction in systolic and diastolic blood pressures were observed (Table 2).

**Table 2:** Longitudinal changes of metabolic variables across the three glycaemic response group (data are mean change ± standard error).

	Good glycaemic responders	Moderate glycaemic responders	Glycaemic non-responders
HbA1c (%)	−2.04∗ ± 0.04	−0.5∗ ± 0.05	0.64∗ ± 0.07
HbA1c (mmol/mol)	−22.2∗ ± 0.5	−5.5∗ ±0.5	6.9∗ ± 0.7
BMI (kg/m^2^)	−1.7∗ ± 0.1	−1.0∗ ± 0.1	−0.7∗ ± 0.2
Waist circumference (cm)	−4.5∗ ±0.5	−2.7∗ ± 0.6	−1.8^#^ ± 0.8
Fasting Blood Glucose (mmol/L)	−3.0∗ ± 0.3	−1.3∗ ± 0.3	0.1 ± 0.4
ALT (μ/L)	−7.3∗ ± 1.1	−1.4 ± 1.3	2.2 ± 1.7
GGT (μ/L)	1.8 ± 1.1	4.9∗ ± 1.2	5.7∗ ± 1.7
Platelets (× 10^9^/L)	38.0∗ ± 2.1	32.7∗ ±2.3	34.8∗ ± 3.2
Fib-4	−0.23∗ ± 0.04	−0.14∗ ± 0.04	−0.13^#^ ± 0.05
FLI	−6.5∗ ± 0.6	−2.7∗ ± 0.7	−0.9∗ ± 0.9
SBP (mmHg)	−3.5∗ ± 1.0	−5.1∗ ± 1.1	−3.0^#^ ± 1.5
DBP (mmHg)	−2.8∗ ± 0.7	−3.0∗ ± 0.7	−2.5^#^ ± 1.0
Creatinine (μmol/L)	2.6 ± 1.3	2.6^#^ ± 1.5	1.8 ± 2.0
Total Cholesterol (mmol/L)	−0.31∗ ± 0.06	−0.14^#^ ± 0.07	−0.06 ± 0.10

^#^p < 0.05 compared to baseline, repeated measures ANOVA.

∗p < 0.001 compared to baseline, repeated measures ANOVA.

*Abbreviations:* HbA1c, glycated haemoglobin, BMI, body mass index, ALT, alanine-transferase, GGT, gamma-glutamyl transferase, FLI, fatty live index, Fib-4, Fibrosis-4 score, SBP, systolic blood pressure, DBP, diastolic blood pressure.

After 1 year of treatment, transitions across FLI classes of risk are described in Table 5. A multiple linear regression model for change in HbA1c showed a significant association with the change in FLI class (R = 0.706, R^2^ = 0.498, p < 0.001, Decreased – Increased, estimate 0.73, p = 0.03) after adjustment for change in BMI, age, class of drug, and baseline values of HbA1c and FLI (Table 4).

A multiple linear regression analysis showed a significant correlation between the changes in FLI and glycated haemoglobin after adjustment for change in BMI, age, sex, drug class and FLI at baseline (R = 0.468, overall model test, p = 0.001, ANOVA for change in HbA1c p = 0.007) (Fig. 1b) (Table 5). Change in BMI and age significantly correlated with change in FLI, whereas agent choice did not seem to have an impact in this regression model.

**Figure 2:**
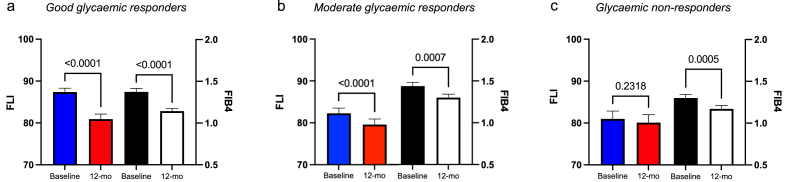
The greatest improvement in FLI was observed in the *good responders* (ΔHbA1c > 11 mmol/mol) compared to the *moderate* (11 < ΔHbA1c < 0 mmol/mol) and *non-responders* (ΔHbA1c < 0 mmol/mol) (a–c).

Those patients who responded well to new drug initiation, *good glycaemic responders*, had a significant reduction in FLI (87.3 *vs.* 80.9; baseline *vs*. 12 months, p < 0.0001). *Moderate glycaemic responders* had a smaller reduction in FLI (82.3 vs 79.6; baseline *vs*. 12 months, p < 0.0001). There was no significant change in FLI in the *glycaemic non-responders* (79.0 vs 80.1; baseline *vs*. 12 months, p = 0.23) (Fig. 2a–c).

**Figure 3:**
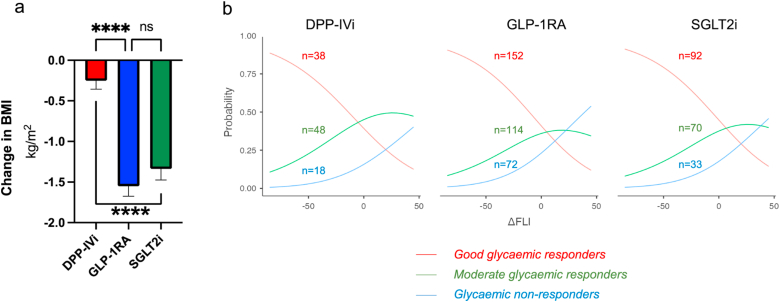
(a) BMI decreased significantly in those patients treated with GLP-1RA and SGLT2i, but not DPP-IVi. (b) Multiple logistic regression demonstrated that the probability of FLI improvement was highest in the good-responders and that this was similar across all 3 classes of glucose lowering agent.

BMI significantly decreased after 12 months of treatment in GLP-1RA (−1.55 ±0.11 kg/m^2^, p < 0.0001) and SGLT2i (−1.34 ± 0.14 kg/m^2^, p < 0.0001) groups, but not in the DPP-IVi (−0.25 ±0.20 kg/m^2^, p = 0.788) group. BMI changes in the SGLT2i and GLP-1RA treated groups were similar (p = 0.46) but differed significantly when compared to patients treated with DPP-IVi (p < 0.001) (Fig. 3a). Logistic regression analysis stratified by glycated haemoglobin response over time and adjusted for ΔBMI, sex, age, FLI at baseline and drug classes, demonstrated a significant association with change in FLI (p < 0.001). Improvements in FLI were most likely in *good glycaemic responders* whilst worsening of FLI was more likely in *moderate* and *non-responders*. The patterns of predicted FLI response to changes in glycated haemoglobin were similar across all 3 classes of agent (Fig. 3b).

Transitions across FIB-4 classes of risk are described in Table 3. The same model used for change in FIB-4 class was applied to the study of change in FLI class and the association was not significant (Table 4).

**Table 3:** Proportions and absolute numbers of patients transitioning between FLI and FIB-4 classes.

		**Good responders**				**Moderate responders**				**Non responders**		

*FIB-4 Class*		Baseline (% (n))		12 mo (% (n))		Baseline (% (n))		12 mo (% (n))		Baseline (% (n))		12 mo (% (n))
Low		56 (159)		73 (206)		44 (102)		64 (148)		57 (70)		70 (86)
Indeterminate		40 (113)		24 (69)		52 (120)		34 (78)		43 (52)		30 (37)
High		4 (10)		3 (7)		4 (10)		2 (6)		0 (1)		0 (0)

		**Good responders**				**Moderate responders**				**Non responders**		

*FLI Class*		Baseline (%(n))		12 mo (%(n))		Baseline (%(n))		12 mo (%(n))		Baseline (%(n))		12 mo (%(n))
Low		1 (3)		5 (13)		3 (7)		3 (8)		3 (4)		4 (5)
Indeterminate		5 (15)		10 (29)		9 (21)		14 (31)		16 (19)		16 (19)
High		94 (264)		85 (240)		88 (204)		83 (193)		81 (100)		80 (99)

At baseline, there was no relationship between glycated haemoglobin and Fib-4 (Fig. 1c, R = 0.133, overall model test p = 0.045, ANOVA for HbA1c at baseline p = 0.289). However, mirroring our observations with FLI, we observed a positive correlation between change in glycated haemoglobin and Fib-4 (after adjustment for change in BMI, sex, age, drug classes and Fib-4 at baseline) (R = 0.666, overall model test p = 0.001, ANOVA for change in HbA1c p = 0.037) (Fig. 1d) (Table 4). In this multiple linear regression model, drug class but not change in BMI was associated with change in Fib-4. Adding change in waist circumference to the model did not change the outcome in terms of correlation between change in FLI and change in HbA1c. A significant reduction in Fib-4 was observed in all groups with the greatest decrease in the *good glycaemic responders* (1.37–1.14, p < 0.001) (Table 2).

**Table 4:** Multiple linear regression modeling to determine the impact of change in HbA1c in change of FLI class. This model includes adjustment for change in BMI, Age, class of drugs HbA1c at baseline and FLI class at baseline.

Multiple Linear Regression Model for ΔHbA1c R = 0.706, R^2^ = 0.498, p < 0.001∗			
*Predictor*	Estimate	SE	P value^#^
Change of FLI class:			
Increased – Decreased	0.73	0.33	0.03
Stable – Decreased	0.20	0.13	0.147
ΔBMI	0.12	0.02	<0.001
Age	0.001	0.004	0.652
Class:			
GLP-1RA – DPP-IVi	0.1	0.11	0.395
SGLT-2i – DPP-IVi	0.22	0.12	0.06
HbA1c baseline	−0.70	0.03	<0.001
FLI Class baseline:			
Indeterminate – Low	0.04	0.28	0.872
High – Low	−0.07	0.26	0.796
Multiple Linear Regression Model for ΔHbA1c R = 0.705, R^2^ = 0.497, p < 0.001∗			
Change of FIB-4 class:			
Increased – Decreased	−0.06	0.17	0.72
Stable – Decreased	−0.11	0.11	0.293
ΔBMI	0.13	0.02	<0.001
Age	0.004	0.004	0.287
Class:			
GLP-1RA – DPP-IVi	0.04	0.11	0.712
SGLT-2i – DPP-IVi	0.16	0.12	0.178
HbA1c baseline	−0.70	0.03	<0.001
FIB-4 Class baseline:			
Indeterminate – Low	0.43	0.21	0.042
High – Low	0.58	0.23	0.012

∗P-values for overall model test.

^#^Omnibus test ANOVA for each variable.

**Table 5:** Multiple linear regression modeling to determine the impact of baseline FLI and Fib-4 respectively, age, sex, glucose lowering agent, change in BMI and change in glycated haemoglobin on fatty liver index (FLI) and FIB-4. ∗P-values for overall model test. ^#^Omnibus test ANOVA for each variable.

Multiple Linear Regression Model for ΔFLI R = 0.468, R^2^ = 0.219, p < 0.001∗			
Predictor	Estimate	SE	P^#^
Sex: M− F	−0.703	0.788	0.373
Class: GLP1-RAs – DPP4is	0.057	1.195	0.962
Class: SGLT2is – DPP4is	−1.363	1.210	0.260
Age	−0.106	0.038	0.005
ΔBMI	2.283	0.198	<0.001
ΔHbA1c	0.834	0.307	0.007
FLI baseline	−0.004	0.024	0.852
Multiple Linear Regression Model for ΔFib-4 R = 0.666, R^2^ = 0.443, p < 0.001∗			
Sex: M− F	−0.025	0.0376	0.721
Class: GLP1-RAs – DPP4is	−0.234	0.052	0.001
Class: SGLT2is – DPP4is	−0.173	0.055	0.002
Age	−0.014	0.002	0.001
ΔBMI	−0.005	0.009	0.585
ΔHbA1c	0.030	0.014	0.037
Fib-4 baseline	−0.629	0.029	0.001

## 4 Discussion

In this *post-hoc* analysis of clinical data, we have demonstrated a robust cross-sectional relationship between glycated haemoglobin and FLI as well as Fib-4, that is independent of BMI. Furthermore, our longitudinal data suggest that reductions in glycated haemoglobin improve NAFLD independently both of any change in BMI and of the use of specific glucose-lowering agents.

There are several retrospective reports in the published literature, that have demonstrated an association between NAFLD severity (using liver chemistry, non-invasive surrogate biomarkers as well as liver histology on biopsy) and glycaemic control in patients with type 2 diabetes [17,[22], [23], [24], [25], [26]]. However, due to the retrospective or cross-sectional nature of these studies, it is difficult to draw any conclusions regarding the causality of elevated blood glucose/HbA1c and severity of NAFLD. Recently, Mantovani et al. [27] reported a significant worsening of HbA1c after 5 years observation in a group of post-menopausal women with significant fibrosis at baseline that was independent of glucose lowering treatment, BMI and baseline HbA1c. These observations are in keeping with a bidirectional relationship between glucose control and NAFLD progression.

Published data looking at the longitudinal impact specifically of alterations in glycemic control and its impact on NAFLD are sparse. Two prospective studies have demonstrated that the addition of insulin (compared to GLP-1RA) can improve liver fat content, and this was associated with a reduction in glycated haemoglobin [[12], [13], [14]]. Unusually in these studies, the introduction of insulin treatment was not associated with significant weight gain. Moreover, explanations regarding the mechanisms behind liver fat content reduction in the group of patients treated with insulin were not provided.

There is now an emerging body of evidence suggesting that GLP-1RA and SGLT2 inhibitors have a beneficial impact in NAFLD, whereas DPP-IV inhibitors seem to have a near-null effect [[28], [29], [30]]. However, both SGLT2i [31] and GLP-1RA [32] cause significant weight loss alongside improvements in blood glucose control and dissecting the relative contributions of both of these mechanisms to the improvement in NAFLD have not been systematically addressed. The potential lack of benefit of DPP-IVi may relate to the fact that studies were undertaken in individuals where there was excellent glycaemic control (or in patients without diabetes) [33]. To our knowledge, only one study [34] reported a reduction in intrahepatic fat content in patients with T2D treated with sitagliptin. Interestingly, in people without diabetes, SGLT2i do not seem to improve hepatic steatosis [35]. However, the SGLT2i, Empagliflozin, when compared to placebo, provided a significant 34% reduction in liver fat content in a cohort of T2D patients. In this study, 5% weight loss was achieved only in the 27% of patients, and this could suggest additional mechanisms of liver fat reduction that might include a direct contribution of glucose lowering [29].

There are several plausible mechanisms by which improvements in glycaemic control might improve NAFLD. Glucose is the substrate for *de novo* lipogenesis (DNL) and it is well described that in patients with diabetes and NAFLD, DNL forms a major contribution to hepatic triglyceride accumulation. Improving glycemic control would limit the availability of glucose as substrate for hepatic DNL. Glucose is also able to drive the activation of stellate cells that are central to the fibrotic response in NAFLD [36] and therefore has the potential to modulate the fibrotic response to the lipid and inflammatory insults that are key drives to advanced NAFLD.

A modest reduction in Fib-4 was observed in all groups with the greatest reduction in the *good glycaemic responders*. Clinical relevance of this observation requires further investigations. The 12-month duration of intervention in this study is a short time in which to see significant changes and only a single surrogate marker of fibrosis risk (Fib-4) was used. However, studies using antiviral agents for the treatment of chronic hepatitis C have shown a reduction in advanced fibrosis after at least 12 months of treatment, but long-term data on fibrosis remission are lacking [37].

There are some limitations in the current analysis. The FLI is a surrogate marker for fatty liver screening and diagnosis [38] and does not allow a direct quantification of hepatic steatosis. In addition, the retrospective collection of data may lead to a patient selection bias for the initiation of specific glucose lowering agents. Differences at baseline (Table 1) could also have influenced the outcomes. However, our statistical modelling approach based on regression analyses, takes into account baseline variability. Including the adjustment for baseline variability, there remained a robust association between glycaemic control and improvement in FLI.

In conclusion, whilst weight loss must be the key priority in treating patients with NAFLD, these data suggest that optimization of glycaemic control independently of weight should be an important part of the management of patients with NAFLD. However, they do not replace the need for prospective, well-designed, randomized controlled studies to specifically address the quantitative impact of optimization of glucose control in patients with NAFLD.

## Disclosure summary

JWT has been an advisory board member for Novo Nordisk, Pfizer, Poxel and Lumos. GDT has received support from Novo Nordisk, Lilly, Abbott, Johnson and Johnson, Ascensia, Takeda, Becton Dickinson and Sanofi. JWT and GDT are supported by the National Institute for Health Research (NIHR) Oxford Biomedical Research Centre (BRC). The views expressed are those of the authors and not necessarily those of the NHS, the NIHR or the Department of Health.

## Financial support

S.C. Visiting Clinical Fellowship is funded by EZ Funder's Scholarship Program.
