# Supplementary material for: Improved glycaemic control in patients with type 2 diabetes has a beneficial impact on NAFLD, independent of change in BMI or glucose lowering agent
Source: Nutr Metab Cardiovasc Dis. 2023 Mar;33(3):640–8. doi: 10.1016/j.numecd.2022.12.010 (PMC11876092; doi:10.1016/j.numecd.2022.12.010)
Supplement: Multimedia component 1 [file mmc1.docx]

**Supplementary Materials**

**Improved glycaemic control in patients with type 2 diabetes has a beneficial impact on NAFLD, independent of change in BMI or glucose lowering agent.**

**Running Title:** Improved glycaemic control and NAFLD

**Authors**: Santo Colosimo^1,2^, Garry D. Tan^2^, Maria Letizia Petroni^3^, Giulio Marchesini^3^ and Jeremy W. Tomlinson^2^

**Table S1**. Effect of statin use on FLI change across groups of glycemic responders.

| ANOVA for ΔFLI  Post-hoc HbA1c response | | | |
| --- | --- | --- | --- |
| Responders | STATIN/EZETIMIBE | NO STATIN | P^a^ |
| GOOD | -6.33 | -5.12 | 0.285 |
| MODERATE | -3.41 | -2.21 | 0.335 |
| NON-RESPONDERS | -1.11 | -0.45 | 0.700 |

^a^ No change in significance after Bonferroni or Tukey correction.

**Table S2**. Effect of statin use on FIB-4 change across groups of glycemic responders.

| ANOVA for ΔFIB-4  Post-hoc HbA1c response | | | |
| --- | --- | --- | --- |
| Responders | STATIN/EZETIMIBE | NO STATIN | P^a^ |
| GOOD | -0.22 | -0.24 | 0.703 |
| MODERATE | -0.09 | -0.17 | 0.382 |
| NON-RESPONDERS | -0.14 | -0.10 | 0.764 |

^a^ No change in significance after Bonferroni or Tukey correction.
